# Function and Evolution of the Sox Multienzyme Complex in the Marine Gammaproteobacterium *Congregibacter litoralis*

**DOI:** 10.1155/2014/597418

**Published:** 2014-03-31

**Authors:** Stefan Spring

**Affiliations:** Leibniz Institute DSMZ-German Collection of Microorganisms and Cell Cultures, Inhoffenstraße 7B, 38124 Braunschweig, Germany

## Abstract

Core sets of *sox* genes were detected in several genome sequenced members of the environmental important OM60/NOR5 clade of marine gammaproteobacteria. However, emendation of media with thiosulfate did not result in stimulation of growth in two of these strains and cultures of *Congregibacter litoralis* DSM 17192^T^ did not oxidize thiosulfate to sulfate in concentrations of one mmol L^−1^ or above. On the other hand, a significant production of sulfate was detected upon growth with the organic sulfur compounds, cysteine and glutathione. It was found that degradation of glutathione resulted in the formation of submillimolar amounts of thiosulfate in the closely related *sox*-negative strain *Chromatocurvus halotolerans* DSM 23344^T^. It is proposed that the Sox multienzyme complex in *Congregibacter litoralis* and related members of the OM60/NOR5 clade is adapted to the oxidation of submillimolar amounts of thiosulfate and nonfunctional at higher concentrations of reduced inorganic sulfur compounds. Pelagic bacteria thriving in the oxic zones of marine environments may rarely encounter amounts of thiosulfate, which would allow its utilization as electron donor for lithoautotrophic or mixotrophic growth. Consequently, in evolution the Sox multienzyme complex in some of these bacteria may have been optimized for the effective utilization of trace amounts of thiosulfate generated from the degradation of organic sulfur compounds.

## 1. Introduction

Aerobic marine gammaproteobacteria affiliated to the OM60/NOR5 clade are widespread in saline environments and of ecological importance in several euphotic coastal environments [1]. It is thought that aerobic anoxygenic photoheterotrophy provides some members of this clade with a selective advantage against competing obligate chemoheterotrophic bacteria [2]. Besides light energy, the oxidation of reduced inorganic sulfur compounds to sulfate is utilized by a large number of heterotrophic proteobacteria as energy yielding process for mixotrophic growth. Several pathways for the oxidation of reduced sulfur compounds to sulfate are known in bacteria, but most knowledge exists about a thiosulfate oxidizing multienzyme complex, which is encoded by a set of sulfur oxidizing (*sox*) genes [3]. It was found that the genes* soxA, B, C, D, X, Y, *and* Z* are present in most, if not all, bacteria that are able to oxidize thiosulfate to sulfate without forming a free intermediate [4, 5]. Hence, a common mechanism for the direct oxidation of thiosulfate to sulfate encoded by* sox* genes in bacteria is discussed. In a previous study the distribution of* sox* genes among members of the OM60/NOR5 clade was revealed by analyses of sequenced genomes and detection of the* soxB* gene (representing the key enzyme sulfate thiohydrolase) with specific PCR primers [6]. It turned out that* sox* genes are present mainly in members of the OM60/NOR5 clade that encode also genes enabling aerobic anoxygenic photoheterotrophy, like* Congregibacter litoralis *(*C. litoralis*) DSM 17192^T^,* Congregibacter* sp. strain NOR5-3,* Luminiphilus syltensis* DSM 22749^T^, or the isolate HTCC2080. However, there is no stringent correlation of genes encoding Sox proteins and subunits of the photosynthetic apparatus, because the isolate IMCC3088 encodes* sox* genes, but no photosynthetic apparatus [7], whereas the bacteriochlorophyll* a*-containing strains* Pseudohaliea rubra* DSM 19751^T^ and* Chromatocurvus halotolerans* DSM 23344^T^ do not encode a* soxB* gene representing a Sox multienzyme complex. Recently, it was shown that in* C. litoralis *emendation of cultivation media with thiosulfate did not stimulate growth [8]. This was unexpected, because in marine members of the* Roseobacter *clade that encode a complete set of* sox* genes mixotrophic growth with thiosulfate as additional energy source could be demonstrated [9]. Hence, aerobic marine bacteria may benefit from* sox* genes in several ways that are independent of the well-known lithotrophic oxidation of thiosulfate to sulfate. To get a clue about a yet unknown function of* sox *genes in aerobic marine gammaproteobacteria a study was initiated in which the sulfur metabolism in* C. litoralis* was analyzed in detail and compared with closely related species lacking a Sox complex.

## 2. Materials and Methods

### 2.1. Used Strains and Cultivation Conditions

The following type strains were used in this study and taken from the collection of the Leibniz Institute DSMZ (Braunschweig, Germany):* C. litoralis* DSM 17192^T^,* Luminiphilus syltensis* DSM 22749^T^,* Chromatocurvus halotolerans* DSM 23344^T^, and* Pseudohaliea rubra* DSM 19751^T^. For routine cultivation all strains were grown in SYPHC complex medium [6] under air atmosphere at 28°C. The preparation of defined marine media and the generation of distinct gas atmospheres for growing strains in batch cultures under semiaerobic incubation conditions have been described elsewhere [6, 10]. The SYPHC complex medium and defined marine medium contained 35.0 g L^−1^ sea salts (Sigma S9883) resulting in a sulfate concentration of around 25 mM. The growth and sulfate production of* C. litoralis* and* Chromatocurvus halotolerans* with various sulfur compounds were determined in a carbonate-buffered saline medium devoid of sulfate. The basal salt solution was named SF and had the following composition (per liter demineralized water): 21.0 g NaCl, 2.5 g MgCl_2_  × 6 H_2_O, 1.0 g KCl, 0.2 g CaCl_2_  × 2 H_2_O, 0.1 g NH_4_Cl, 0.05 g KH_2_PO_4_, 2.5 g NaHCO_3_, and 1 mL vitamins solution (see DSMZ medium 503). The vitamins, KH_2_PO_4_, NaHCO_3_, and any additional substrates were added to the basal medium after autoclaving from stock solutions sterilized by filtration. The pH of the completed medium was adjusted to pH 7.5. In most experiments a sulfate-poor medium designated LS was used that was obtained by transferring an inoculum size of 1 vol% from defined marine medium to SF medium resulting in an initial sulfate concentration of around 250 µM, which was sufficient to prevent growth inhibition by sulfate limitation. Cultures in essentially sulfate-free media were obtained by two successive transfers in SF medium containing L-glutamate as carbon source. All used chemicals were obtained from Sigma-Aldrich (Taufkirchen, Germany) and complex nutrients from DIFCO BBL (Becton Dickinson; Heidelberg, Germany).

### 2.2. Determination of Growth and Quantification of Sulfur Compounds

The absorbance values of growing cultures were determined in a Thermo Scientific BioMate 6 split beam UV/visible spectrophotometer using 1 cm light path disposable cuvettes and water as blank. Sulfate concentrations were determined in cultures of* C. litoralis* and* Chromatocurvus halotolerans* grown in LS or SF medium by the barium chloride turbidity method of Madsen and Aamand [11]. Uninoculated controls were incubated in parallel and used to determine a possible chemical production of sulfate. Values below 50 µM sulfate could not be exactly determined with this method due to a nonlinearity of the slope of the barium sulfate assay in this concentration range. Thiosulfate concentrations were estimated in culture supernatants by the cyanolysis method as described by Kelly et al. [12] and sulfite was quantified by the method proposed by Denger et al. [13].

### 2.3. Semiquantitative Detection of soxB Transcripts Using PCR

RNA was extracted from early stationary phase cultures of* C. litoralis* DSM 17192^T^ grown under various conditions using the RNeasy Midi kit of Qiagen (Hilden, Germany) as described previously [2]. Reverse transcriptase-PCR (RT-PCR) of mRNA was performed with the OneStep RT-PCR kit of Qiagen following the instructions given by the manufacturer and using 0.5 µg of RNA. For the semiquantitative detection of transcripts of the* C. litoralis soxB* gene the forward primer KT71 soxB-F (5′-TCCAGGCGATAGTTGAATCC-3′) and the reverse primer KT71 soxB-R (5′-AGCTTCGACCAGCTCATTGT-3′) were used. The resulting PCR product had a size of 290 bp. Amplification of transcripts, visualization of PCR products, and normalization of mRNA levels were done as reported previously [2].

### 2.4. Phylogenetic Analyses of Sox Proteins

Amino acid sequences of the proteins SoxB, SoxC, and SoxA were obtained from the UniProt database (release 2013_08) and aligned using the ClustalW algorithm implemented in the ARB package [14]. Phylogenetic trees based on aligned protein sequences were reconstructed using the ARB distance matrix (neighbor-joining) program with the PAM correction and maximum likelihood (RAxML) program with the PROTGAMMA option and BLOSUM62 as amino acid substitution model. No filter or weighting masks were used to constrain the used positions of the alignment.

## 3. Results and Discussion

### 3.1. Effect of Thiosulfate on the Growth Response of Members of the OM60/NOR5 Clade

**Figure 1:**
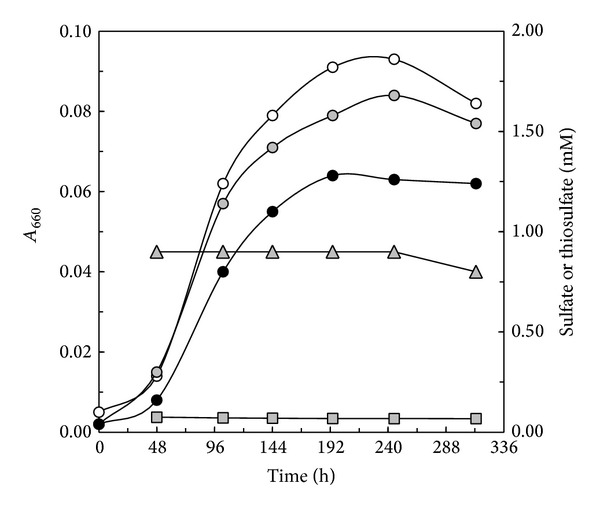
Growth response of* C. litoralis* in LS medium containing 2.5 mM L-glutamate as carbon source and supplemented with various amounts of thiosulfate. Batch cultures were incubated in the dark under semiaerobic conditions. Growth over time determined as* A*_660_ values is indicated by open circles (no added thiosulfate), circles filled in grey (1 mM thiosulfate), and closed circles (10 mM thiosulfate). The concentrations of thiosulfate (triangles filled in grey) and sulfate (squares filled in grey) were monitored in cultures growing with 2.5 mM L-glutamate and 1 mM thiosulfate.

The growth response of the type strains of* C. litoralis* and* Luminiphilus syltensis* both encoding a core set of* sox* genes (*soxA, B, C, D, X, Y, and Z*) and the* sox*-negative strains* Chromatocurvus halotolerans* DSM 23344^T^ and* Pseudohaliea rubra* DSM 19751^T^ was tested in defined and complex marine media with and without thiosulfate under various incubation conditions. Some representative results are shown in Table 1 and indicate that thiosulfate had no stimulatory effect on the growth of the tested strains. Thiosulfate rather had a growth inhibiting effect that was even more pronounced in strains encoding a Sox complex. In a previous study it was assumed that the absence of growth stimulation by thiosulfate in cultures of* C. litoralis* could be due to the missing of putative essential* sox *genes, like, for example,* soxV*, which are present in several lithotrophic thiosulfate oxidizers and may be responsible for the coupling of thiosulfate oxidation with energy generation [8]. In order to reveal if thiosulfate is actually oxidized to sulfate by* C. litoralis* it was necessary to determine sulfate concentrations in spent culture fluid. Hence, defined media with salinity close to sea water but devoid of sulfate were designed to enable measurement of small amounts of produced sulfate without the high background values of typical marine media containing around 20–25 mM of sulfate. It was found that a concentration of 250–400 µM sulfate in cultivation media was optimal for this purpose, because on the one hand growth was not limited by the availability of a sulfur source and on the other hand the used concentration did not interfere with the applied barium chloride turbidity method used for sulfate determination. In the remainder of this study cultivation media containing sulfate concentrations below 400 µM are designated as low sulfate concentration (LS) media. Representative growth curves of* C. litoralis* in sulfate-poor LS media without thiosulfate or supplemented with 1 mM and 10 mM thiosulfate indicate that the growth inhibiting effect of thiosulfate increased with its concentration and that no significant conversion of thiosulfate to sulfate took place (Figure 1). Thus, it can be concluded that under the applied cultivation conditions the Sox multienzyme pathway was not active in cells of* C. litoralis*. It has to be noted that the chemical determination of sulfate and thiosulfate concentrations during growth was not feasible in cultures containing 10 mM thiosulfate, because thiosulfate in such high amounts interfered with the barium chloride method of sulfate estimation. A negative effect of high thiosulfate concentrations on bacterial growth was observed previously also in the mixotrophic proteobacteria* Thiobacillus* strain Q [15] and* Limnobacter thiooxidans* [16], as well as in the lithotrophic gammaproteobacterium* Acidithiobacillus* (formerly* Thiobacillus) thiooxidans *[17]. In* Thiobacillus* strain Q and* Limnobacter thiooxidans* a reduction of growth yield above concentrations of 7 and 20 mM thiosulfate, respectively, correlated with a decrease in sulfate production, which may be due to the formation of inhibitory levels of sulfite [15]. However, this was not the case in* Acidithiobacillus thiooxidans* leading to the assumption that high concentrations of thiosulfate may inhibit growth in this species by having a chelating effect on the cell membrane [17].

### 3.2. Alternative Functions of the Sox Multienzyme Complex

**Figure 2:**
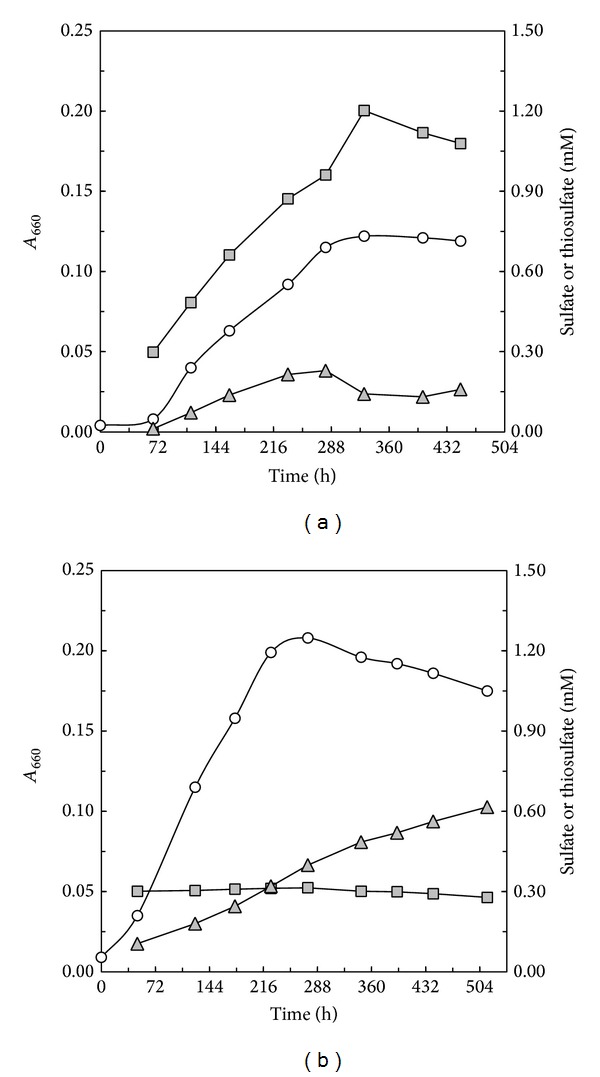
Growth response of* C. litoralis* and* Chromatocurvus halotolerans* in LS medium containing 2 mM L-glutathione as carbon source. Batch cultures of* C. litoralis* (a) and* Chromatocurvus halotolerans* (b) were incubated in the dark under semiaerobic conditions. Growth over time determined as* A*_660_ values is indicated by open circles. Concentrations of thiosulfate and sulfate are indicated by triangles and squares filled in grey, respectively.

To determine if the Sox multienzyme complex could have a function in sulfur metabolism other than mixotrophy the growth response and sulfate production in cultures of* C. litoralis* were tested in sulfate-free (SF) media containing various organic sulfur compounds and glutamate as principal carbon source. Experiments were designed in such a way that substantial growth became only evident in the presence of sulfur compounds, which can be either assimilated or oxidized to sulfate thereby representing a source of sulfur. It has been shown previously that* C. litoralis* can use sulfate as sole source of sulfur and encodes all necessary genes for its assimilation by the APS/PAPS pathway [8]. A significant growth and sulfate production were only observed with the sulfur-containing amino acids L-cysteine and L-cystine, whereas no growth or sulfate production was detected with the following sulfur-containing compounds: dimethyl sulfide, dimethyl disulfide, dimethyl sulfoxide, methanesulfonate, thiourea, thioglycolate, 3,3′-dithiodipropionate, L-methionine, and L-cysteate. In further experiments a production of sulfate could be also observed in cultures of* C. litoralis* in sulfate-free medium if L-cysteine and L-glutamate were replaced with reduced L-glutathione or oxidized (−)-glutathione as the sole carbon source. Glutathione is a tripeptide consisting of the three amino acids L-glutamic acid, L-cysteine, and DL-glycine, in which the amino group of cysteine is linked to the *γ* carboxyl group of glutamic acid by an unusual peptide bond. The advantage of glutathione as substrate compared to the combination of L-cysteine with L-glutamate is that growth is coupled to a single carbon source, so that a potential biphasic growth based on the preferred utilization of glutamate by* C. litoralis* is avoided. In Figure 2(a) a representative growth curve of* C. litoralis* with L-glutathione as substrate reveals that during growth in LS medium sulfate is produced and thiosulfate seems to accumulate transiently. Such a pathway would be not unique, since formation of sulfate with thiosulfate as assumed intermediate product was also observed during the growth of an* Alcaligenes* sp. with mercaptosuccinate as sole source of carbon, sulfur, and energy [18]. Although these results point to an involvement of the Sox complex in the degradation of sulfur-containing amino acids, alternative mechanisms may be also possible. Hence, in a control experiment the* sox*-negative strain* Chromatocurvus halotolerans* was grown in the same sulfate-poor medium with L-glutathione as substrate. As shown in Figure 2(b) it turned out that indeed thiosulfate accumulated without the accompanying production of sulfate, which suggests that thiosulfate is the substrate for sulfate production in cultures of* C. litoralis* growing on glutathione. In sterile media containing cysteine or glutathione no production of sulfate could be detected, whereas the accumulation of trace amounts of thiosulfate was observed. These results suggest a hitherto unknown mechanism for the biogenic generation of thiosulfate in certain marine environments, where organic thiols like cysteine, phytochelatins, or glutathione are produced by bacteria and algae and hence may be present in significant amounts during algal blooms [19–21]. Currently, it is unclear which pathway could be involved in the formation of thiosulfate from organic thiols. In the experimental setup used in this study a nonenzymatic formation of thiosulfate during the degradation of glutathione seems to be also possible. Firstly, thiosulfate production in cultures of* Chromatocurvus halotolerans* appears to follow a slow linear kinetic and proceeds even after the strain has stopped growing, which would be more typical for a nonenzymatic reaction (Figure 2(b)). Secondly, small amounts of sulfite (100–200 *µ*M) were detected in the culture supernatant upon growth of this strain on glutathione (data not shown), so that sulfite is likely an intermediate of thiol oxidation that could react with polysulfides resulting from the spontaneous desulfuration of glutathione. The formation of polysulfides in culture media by autoxidation of glutathione or cysteine can be assumed based on the observation of a characteristic faint yellow color that slowly increased over time. This was confirmed by UV/Vis spectra of sterile semiaerobic culture media containing cysteine which displayed an increase of absorbance values at 300 and 370 nm which is typical for polysulfide formation [22] and not observed in oxygen-free medium containing cysteine in its reduced form. Thiosulfate could then form by the chemical reaction of sulfite with polysulfides according the following formula [23]:

(1) $$\begin{array}{c} {{\text{SO}}_{3}}^{2-}+{{\text{S}}_{n}}^{2-}⟶{{\text{S}}_{2}{\text{O}}_{3}}^{2-}+{{\text{S}}_{n-1}}^{2-} \end{array}$$

The possible mechanisms for the generation of sulfite from cysteine in* C. litoralis* and* Chromatocurvus halotolerans* are however still unclear, because genes encoding potential cysteine dioxygenases were not found in genome-sequenced members of the OM60/NOR5 clade. Interestingly, genes encoding TauD-like sulfonate dioxygenases (KT71_05942 and KT71_15756) and TauE-like sulfite exporters (KT71_00305 and KT71_11279) were detected in the genome of* C. litoralis*, so that only genes responsible for the first steps of cysteine oxidation would be missing. Alternatively, these initial reactions could be catalyzed also by a potential multifunctional sulfonate dioxygenase without cysteate as free intermediate, which would explain why this compound is not a suitable substrate for* C. litoralis* [10].

**Figure 3:**
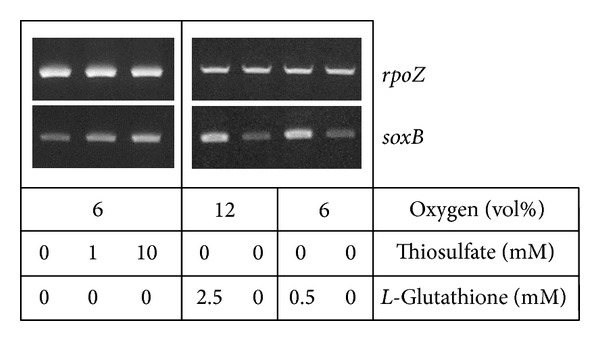
Analyses of the transcription level of the* soxB *gene in* C. litoralis* under various incubation conditions. Cultures were grown in defined marine medium in the dark. The effect of thiosulfate was determined in media containing 2.5 mM L-glutamate as carbon source. The influence of reduced glutathione on* soxB* expression was determined by replacing this substrate with equimolar amounts of the amino acids L-glutamate, L-serine, and DL-glycine. The upper panel shows the results of a RT-PCR of the* rpoZ* gene, which was used to normalize mRNA levels in different samples of extracted RNA. The panel below shows results obtained with the same RNA samples after RT-PCR using specific* soxB* primers.

To demonstrate the actual involvement of a Sox multienzyme complex in the production of sulfate during degradation of glutathione semiquantitative RT-PCR was used to estimate expression of* sox* genes in* C. litoralis*. As shown in Figure 3, a significant increase (ca. 1.7-fold) in the expression level of the* soxB* gene could be demonstrated upon growth of* C. litoralis* in defined marine media containing glutathione as sole carbon source compared to media containing a mixture of the amino acids L-glutamate, L-serine, and DL-glycine. This mixture was chosen, because it represents a highly similar carbon source in which only the sulfhydryl group of the glutathione-bound cysteine is replaced by the hydroxyl group of serine. The stimulation of* soxB* expression could be repeatedly observed, despite variations of the concentration of oxygen in the head space gas atmosphere or substrate concentration (Figure 3). In a complementary experiment the effect of thiosulfate on* soxB* expression was determined in defined marine medium containing 2.5 mM L-glutamate as carbon source. Although a slight induction of* soxB* transcription was found in medium supplemented with 10 mM thiosulfate, it appears that the expression level was lower compared to cultures growing with glutathione.

**Figure 4:**
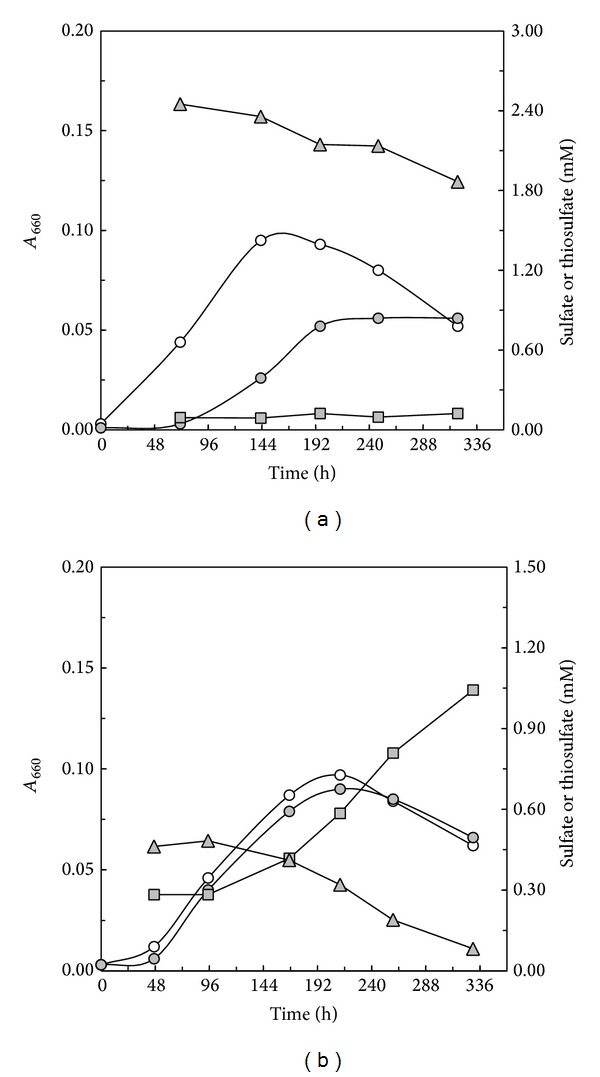
Growth response of* C. litoralis* in LS medium containing 2.0 mM L-glutamate as carbon source compared to medium supplemented with (a) 2.5 mM or (b) 0.5 mM thiosulfate. Batch cultures were incubated in the dark under semiaerobic conditions. Growth over time determined as* A*_660_ values is indicated by open circles (no added thiosulfate) or circles filled in grey (0.5 mM or 2.5 mM thiosulfate). Concentrations of thiosulfate and sulfate measured during growth are shown as triangles and squares filled in grey, respectively.

Thus, multiple lines of evidence suggest that the Sox complex in* C. litoralis* is involved in the degradation of peptide-bound thiol or disulfide groups by the oxidation of thiosulfate that forms as free intermediate. Nevertheless, the question is still open, why the Sox complex in photoheterotrophic members of the OM60/NOR5 clade is inactive against thiosulfate added as additional electron donor for mixotrophic growth. As the* soxB* gene in cells growing with 10 mM thiosulfate was expressed at significant levels, one possible explanation could be that the Sox enzyme complex in this species is optimized for the oxidation of thiosulfate present in submillimolar concentrations and inactive at the amounts of thiosulfate normally used to study mixotrophy, that is, 10–20 mM thiosulfate. This assumption was tested by transferring cells of* C. litoralis* grown on glutathione in LS medium supplemented with either 2.5 mM or 0.5 mM thiosulfate and L-glutamate as carbon source. Degradation of thiosulfate and sulfate production took place only in the culture containing 0.5 mM thiosulfate (Figure 4), thus confirming our conclusion that the Sox multienzyme complex of* C. litoralis* is adapted to the oxidation of small amounts of thiosulfate without any measurable effect on the cellular growth yield, at least in batch cultures. Although, a slight decrease of the thiosulfate concentration is also noted in the culture containing 2.5 mM thiosulfate this is likely attributed to a chemical absorption or degradation, because it was not correlated with the production of sulfate.

### 3.3. Evolution of the Sox Complex in Members of the OM60/NOR5 Clade

#### 3.3.1. Phylogenetic Analyses

**Figure 5:**
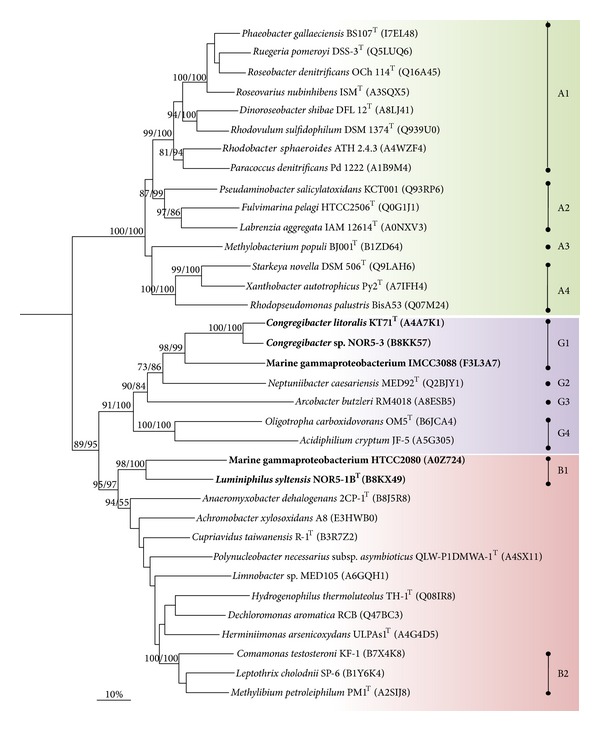
Phylogenetic dendrogram based on full-length protein sequences of SoxB illustrating the positions of members of the OM60/NOR5 clade (in bold). The SoxB sequence of* Thermus thermophilus* HB27 (Q72IT0) was used as outgroup (not shown). The displayed topology of the dendrogram is based on a reconstruction using the neighbor-joining algorithm. Bootstrap values (as percentages of 1000 resampling times) based on neighbor-joining and RAxML calculations are shown in the front of each node; provided that the branching was retrieved with both methods and at least with one reconstruction method, a value of 80% or above was obtained. Type strains are indicated by a superscript T. UniProt accession numbers of the used proteins are given in parentheses. Stable phylogenetic lineages of Sox proteins are indicated by dots or vertical line drawings followed by an arbitrary designation. The shown bar represents an estimated sequence divergence of 10%.

**Figure 6:**
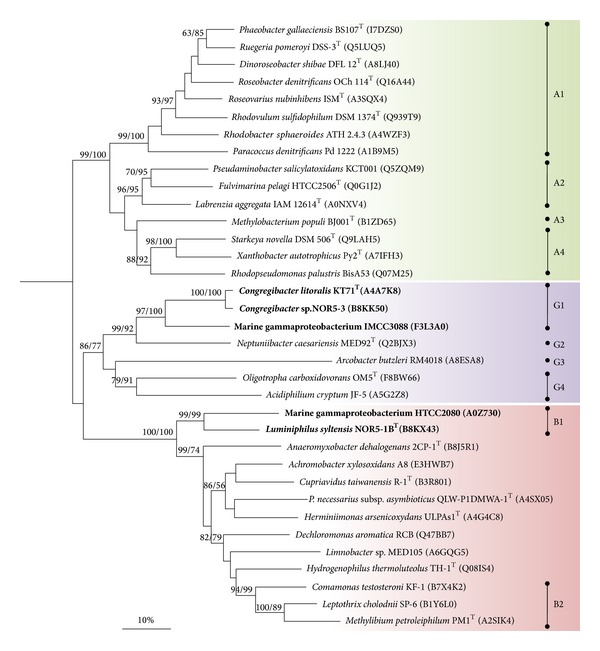
Phylogenetic dendrogram based on full-length protein sequences of SoxC illustrating the positions of members of the OM60/NOR5 clade (in bold). The SoxC sequence of* Thermus thermophilus *HB27 (Q72IT6) was used as an outgroup (not shown). For further details see legend of Figure 5.

**Figure 7:**
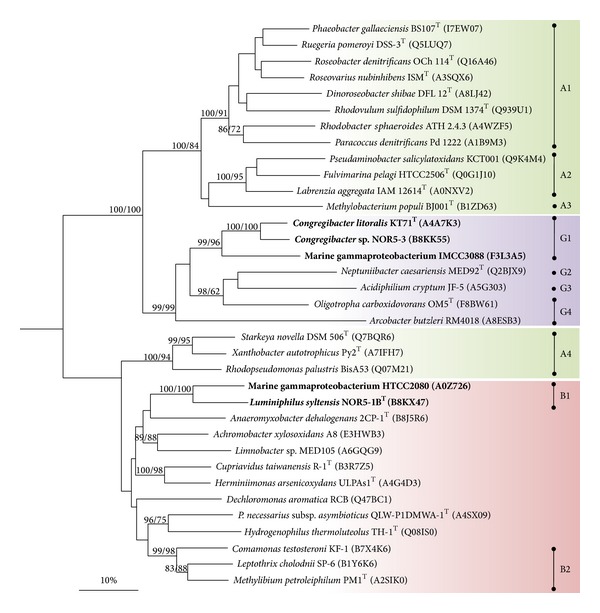
Phylogenetic dendrogram based on full-length protein sequences of SoxA illustrating the positions of members of the OM60/NOR5 clade (in bold). The SoxA sequence of* Thermus thermophilus *HB27 (Q72IT2) was used as an outgroup (not shown). For further details see legend of Figure 5.

In a recent study by Ghosh et al. [24] it is proposed that nonfunctional or atypical Sox complexes in proteobacteria result from a noncongruent evolution of the different modules of the Sox multienzyme complex. In order to determine if this holds true for members of the OM60/NOR5 clade phylogenetic trees were reconstructed using aligned amino acid sequences of the three largest proteins constituting the Sox complex, which are SoxB, SoxC, and SoxA. In order to ensure that only proteins are compared that actually form a Sox multienzyme complex and hence share the same function strains of proteobacteria were selected that encode a complete core set of sox genes organized in a single cluster or operon, which makes participation in a multienzyme complex highly likely, whereas* sox*-like genes dispersed within a genome could have also different functions in metabolism. If several alleles of a distinct* sox* gene were encoded in a genome, the one located within a cluster of the core set of* sox* genes was chosen for a phylogenetic analysis. As a result we could identify three distinct evolutionary lineages of Sox proteins within the Proteobacteria that were essentially obtained in all reconstructed phylogenetic trees (Figures 5, 6, and 7). Lineage A contains almost exclusively alphaproteobacteria affiliated to the* Rhodobacterales* and* Rhizobiales*. Lineage G contains representatives of various phylogenetic groups including* Gamma-*,* Alpha-* and* Epsilonproteobacteria* (*Arcobacter butzleri*), which probably reflects frequent lateral transfer of* sox *genes among members of this lineage. This group comprises also the Sox proteins present in phototrophic purple and green sulfur bacteria that lack the sulfane dehydrogenase SoxCD (data not shown). The third coherent group, lineage B, comprises mainly betaproteobacteria affiliated to the* Burkholderiales* and also deltaproteobacteria (*Anaeromyxobacter dehalogenans*) and members of the OM60/NOR5 clade. This result is in good agreement with the proposed classification of SoxA cytochromes in three phylogenetic groups by Ogawa et al. [25], which therefore corresponds with three major evolutionary lineages of the complete Sox complex.

Based on the presented phylogenetic data it can be deduced that* sox *genes are of polyphyletic origin in members of the OM60/NOR5 clade, in contrast to photosynthesis genes which were derived from a common ancestor [6]. Sox proteins of members of the NOR5-3 and NOR5-4 subclades were affiliated to one coherent cluster of lineage G (G1 in Figures 5–7), which was retrieved with high bootstrap support in all reconstructed trees, whereas Sox proteins of representatives belonging to the environmental important NOR5-1 subclade form a separate cluster within lineage B (B1 in Figures 5–7). The divergent evolution of* sox* operons in members of the OM60/NOR5 clade is also reflected in a different arrangement of core genes: in* Congregibacter *species representing cluster G1 the sequence of genes is* soxCDXYZAKB*, whereas in* Luminiphilus syltensis* and strain HTCC2080 of cluster B1 it is* soxCDYZAXB*. Despite the polyphyletic origin of sox genes in the OM60/NOR5 clade it appears that the different modules of the Sox multienzyme complex evolved in parallel, because the same topology was obtained in reconstructed trees based on the proteins SoxB, SoxC, and SoxA. Thus, shuffling of* sox* genes between strains with a divergent genomic background cannot be the reason for the atypical function of the Sox complex in* C. litoralis* as it was proposed by Ghosh et al. for certain betaproteobacteria [24]. According to their hypothesis the noncongruent evolution of* sox *genes could represent a potential hindrance for the modular protein-protein interactions within the Sox multienzyme complex leading to a nonfunctional or deviant system. However, in our phylogenetic reconstructions shuffling of* sox* genes among members of the* Burkholderiales* could not be underpinned by significant bootstrap values. In contrast, the only potential mosaic systems were detected in some members of the* Rhizobiales* forming the cluster A4 that is shifted from lineage A to B in phylogenetic trees based on SoxA (Figure 7). Interestingly, these species are known to possess a functional Sox multienzyme system allowing generation of energy from thiosulfate oxidation [26–28]. According to the study of Kappler and Maher [29] the three main phylogenetic branches in Figure 7 correspond to SoxA cytochromes of type 1 (A1–A3), type 2 (B1, B2, A4, and unassigned sequences), and type 3 (G1–G4). The three phylogenetic lineages can be correlated with distinguishable structures of SoxA. Enzymes of type 1 are represented by diheme cytochromes, type 2  by monoheme cytochromes, and proteins of type 3 are monoheme cytochromes that require a small binding protein designated SoxK which stabilizes the SoxAX complex. It is noteworthy that the genome of* Starkey novella* encodes a second copy of* soxA* (Snov_1982) that is located in a truncated* sox* operon and represents a diheme SoxA of type 1. Thus, it could be possible that in evolution the complete* sox *operon of* Starkey novella* originally contained a type 1* soxA *gene that was later exchanged by homologous recombination with a type 2* soxA* gene obtained by lateral gene transfer.

#### 3.3.2. Implication for Environmental Genomics

Analyses of metagenomic and single cell genomic libraries revealed a wide distribution of* sox *genes within the microbial community in oxic marine environments [9, 30, 31]. Hence, it can be postulated that the capability to oxidize sulfur compounds via the Sox multienzyme complex is widely spread even among bacteria that thrive in the oxic zones of the oceans where reduced inorganic sulfur compound are present only in negligible amounts, so that their utilization as electron donor for lithotrophic growth appears unlikely. On the other hand, organic sulfur compounds (e.g., dimethylsulfoniopropionate, glutathione, phytochelatins, etc.) are continuously produced by phototrophic primary producers and available as additional substrates for heterotrophic growth especially during algal blooms [19, 32, 33]. In a recent study based on a taxon-specific functional gene microarray it was found that some enzymes of the Sox pathway may be involved in the oxidation of inorganic sulfur compounds during degradation of dimethylsulfoniopropionate [34]. Although the oxidation of small amounts of thiosulfate apparently has no visible effect on the growth response under laboratory conditions, the encoding of* sox *genes must provide members of the OM60/NOR5 clade with a selective advantage; otherwise these genes would not have remained functional. Possible functions could include either a role in detoxification (although at the moment the basis for a growth inhibiting effect of thiosulfate in these bacteria is unknown) or the utilization of thiosulfate as additional reductant. Electrons obtained by the oxidation of thiosulfate could be either channeled in the electron transport chain without visible effect on the growth response due to the low energy yield or may be used for other cellular processes that are not coupled to the generation of energy. The uncoupling of thiosulfate oxidation by the Sox complex with the generation of metabolically useful energy was, for example, observed in cultures of* Hydrogenophilus thermoluteolus* [35].

## 4. Conclusions

The formation of thiosulfate in oxic marine environments is possible by the degradation of cysteine-containing peptides that are part of the particulate organic matter especially during algal blooms. Thiosulfate formed during the utilization of organic thiols is probably mainly available transiently at distinct sites like marine snow aggregates, whereas in suboxic transition zones of sulfide-rich habitats thiosulfate is continuously formed in substantial amounts by chemical processes. Therefore, in marine bacteria several functions of the periplasmic Sox complex could have evolved ranging from the generation of energy during mixotrophic or lithoautotrophic growth to the degradation of trace amounts of thiosulfate during utilization of organic thiols without significant contribution to the cellular energy balance.   For the future, it is planned to generate mutants deficient in* sox* genes in order to obtain more information about the elusive role of the Sox complex in gammaproteobacteria belonging to the environmental important OM60/NOR5 clade.

## Figures and Tables

**Table 1:** Growth response of several type strains of the OM60/NOR5 clade in batch culture with and without thiosulfate.

Strain	Presence of* sox *genes*	Carbon source	Growth yield (*A*_660 nm_)	
			No thiosulfate	10 mM thiosulfate
*C. litoralis* DSM 17192^T^	+	5 mM pyruvate	0.168	0.111
		10 mM pyruvate + 1 g L^−1^ Y.E.	0.676	0.495
*L. syltensis* DSM 22749^T^	+	5 mM pyruvate	0.244	0.208
		10 mM pyruvate + 1 g L^−1^ Y.E.	0.814	0.588
*C. halotolerans* DSM 23344^T^	−	5 mM pyruvate	0.156	0.142
		10 mM pyruvate + 1 g L^−1^ Y.E.	0.871	0.744
*P. rubra* DSM 19751^T^	−	5 mM pyruvate	0.202	0.161
		10 mM pyruvate + 1 g L^−1^ Y.E.	0.407	0.366

Batch cultures grown in the complex medium SYPHC were incubated under air atmosphere, whereas cultures grown in defined marine medium with 5 mM pyruvate as substrate were incubated under semiaerobic conditions. The growth yield was determined as maximum optical density reached in early stationary phase. *Data from Spring et al. 2013 [6].

Y.E.: yeast extract.
